# Heat shock protein 90 modulates cutaneous vasodilation during an exercise‐heat stress, but not during passive whole‐body heating in young women

**DOI:** 10.14814/phy2.14552

**Published:** 2020-08-26

**Authors:** Gregory W. McGarr, Naoto Fujii, Madison D. Schmidt, Caroline M. Muia, Glen P. Kenny

**Affiliations:** ^1^ Human and Environmental Physiology Research Unit University of Ottawa Ottawa ON Canada; ^2^ Faculty of Health and Sport Sciences University of Tsukuba Tsukuba Japan

**Keywords:** heat loss, microcirculation, oestrogen, sex, skin blood flow

## Abstract

Heat shock protein 90 (HSP90) modulates exercise‐induced cutaneous vasodilation in young men via nitric oxide synthase (NOS), but only when core temperature is elevated ~1.0°C. While less is known about modulation of this heat loss response in women during exercise, sex differences may exist. Further, the mechanisms regulating cutaneous vasodilation can differ between exercise‐ and passive‐heat stress. Therefore, in 11 young women (23 ± 3 years), we evaluated whether HSP90 contributes to NOS‐dependent cutaneous vasodilation during exercise (Protocol 1) and passive heating (Protocol 2) and directly compared responses between end‐exercise and a matched core temperature elevation during passive heating. Cutaneous vascular conductance (CVC_%max_) was measured at four forearm skin sites continuously treated with (a) lactated Ringers solution (control), (b) 178 μM Geldanamycin (HSP90 inhibitor), (c) 10 mM L‐NAME (NOS inhibitor), or (d) combined 178 μM Geldanamycin and 10 mM L‐NAME. Participants completed both protocols during the early follicular (low hormone) phase of the menstrual cycle (0–7 days). Protocol 1: participants rested in the heat (35°C) for 70 min and then performed 50 min of moderate‐intensity cycling (~55% VO_2peak_) followed by 30 min of recovery. Protocol 2: participants were passively heated to increase rectal temperature by 1.0°C, comparable to end‐exercise. HSP90 inhibition attenuated CVC_%max_ relative to control at end‐exercise (*p* < .05), but not during passive heating. While NOS inhibition and combined HSP90 + NOS inhibition attenuated CVC_%max_ relative to control for both protocols (all *p* < .05), they did not differ from each other. We show that HSP90 modulates cutaneous vasodilation NOS‐dependently during exercise in young women, with no effect during passive heating, despite a similar NOS contribution.

## Graphical abstract

We evaluated whether HSP90 contributes to NOS‐dependent cutaneous vasodilation during exercise and passive heating in young women. We directly compared these responses at end‐exercise and the matched core temperature elevation during passive heating. We showed that HSP90 modulates cutaneous vasodilation NOS‐dependently during exercise in young women, while there was no effect of HSP90 inhibition during passive heating, despite a similar NOS contribution.

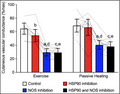

## 1 INTRODUCTION

In human skin, the molecular chaperone, heat shock protein 90 (HSP90), is upregulated by a variety of stressors, including elevations in temperature (Wilson et al., 2000). It is well known that HSP90 binds with and stabilizes the nitric oxide synthase (NOS) enzyme, augmenting nitric oxide production (Harris, Ju, Venema, Blackstone, & Venema, 2000; Ou et al., 2004). We recently showed that HSP90 inhibition attenuated cutaneous vasodilation during an exercise‐heat stress in young men through a NOS‐dependent mechanism (Fujii, Zhang, McNeely, Nishiyasu, & Kenny, 2017). Rodent studies demonstrate that females have lower HSP90 levels relative to males (Voss et al., 2003). In humans, young women also display a smaller NOS contribution to cutaneous vasodilation during local skin heating relative to young men, which may be due to lower HSP90 expression (Stanhewicz, Greaney, Kenney, & Alexander, 2014). However, very little is known specifically about cutaneous vasodilatory control in women during exercise, including the potential modulating effects of HSP90 on this response. As such, it remains unknown if and to what extent HSP90 modulates cutaneous vasodilation in young women during an exercise‐heat stress and whether such a response is NOS‐dependent. Such knowledge is important given growing evidence demonstrating reduced heat dissipation associated with altered cutaneous vasodilation in women relative to men.

We previously showed that the HSP90 contribution to cutaneous vasodilation during an exercise‐heat stress was only evident during the latter stages of steady‐state exercise in young men, when the level of hyperthermia was the highest (i.e., the final 20 min of a 50‐min exercise bout when body core temperature approached a stable elevation of ~1.0°C above baseline rest) (Fujii, Zhang, et al., 2017). Thus, during an exercise‐heat stress there may be a level of hyperthermia (and therefore body core temperature threshold) above which HSP90 is upregulated and thus becomes functionally important in modulating cutaneous vasodilation. However, whether the HSP90 contribution to cutaneous vasodilation during an exercise‐heat stress is due to the increase in body core temperature per se or if it is also associated with exercise‐induced increases in metabolic and hemodynamic strain, remains uncertain. This is important since the mechanisms regulating cutaneous vasodilation in response to body core temperature elevations during passive heating do not always coincide with those observed during an exercise‐heat stress (Fujii et al., 2018; Holowatz, Thompson, & Kenney, 2006; Kellogg, Zhao, & Wu, 2009; McCord, Cracowski, & Minson, 2006; McNamara, Keen, Simmons, Alexander, & Wong, 2014; Meade et al., 2019).

In this study we evaluated responses in young women who were tested during the low‐hormone phase of the menstrual cycle in order to avoid potential confounding effects of sex or menstrual cycle phase on both heat stress protocols. The first purpose of this study was to evaluate the HSP90 contribution to cutaneous vasodilation during an exercise‐heat stress in young women. We hypothesized that HSP90 inhibition would attenuate cutaneous vasodilation NOS‐dependently in young women at end‐exercise when body core temperature was sufficiently elevated (~1°C above baseline rest). Second, to determine whether the HSP90 contribution to cutaneous vasodilation in women is related to an increase in body core temperature per se, we evaluated this response during passive whole‐body heating up to an increase in body core temperature of ~1°C above baseline (equivalent to mean end‐exercise response). We hypothesized that during passive heating, HSP90 inhibition would attenuate cutaneous vasodilation NOS‐dependently in young women when body core temperature was sufficiently elevated. Third, we directly compared the HSP90 contributions to cutaneous vasodilation between an exercise‐induced heat stress and a matched elevation in body core temperature, and therefore thermal drive, during a passive heat stress. We hypothesized that there would be comparable HSP90 contributions to cutaneous vasodilation in young women between end‐exercise and a matched elevation in body core temperature during passive heating.

## 2 MATERIALS AND METHODS

### 2.1 Ethical approval

This study was approved by the University of Ottawa Health Sciences and Science Research Ethics Board (H04‐17‐01) in accordance with the guidelines set forth by the Declaration of Helsinki, with the exception that registration in a database was not done. All volunteers provided verbal and written informed consent prior to participation.

### 2.2 Participants

Eleven habitually active young women (23 ± 3 years) participated in this study. All participants were screened for cardiovascular, respiratory, and metabolic diseases before participating. The participants were nonsmoking, and none were currently taking prescription medications, with the exception of hormonal contraceptives. Participants’ body mass, height, body fat percentage, body surface area, and peak oxygen uptake (VO_2peak_) were (mean ± *SD*) 62.0 ± 11.1 kg, 1.64 ± 0.07 m, 22.3 ± 5.4%, 1.67 ± 0.17 m^2^, and 37.7 ± 3.3 ml/kg min^−1^, respectively.

### 2.3 Experimental design

All participants completed one screening session and two experimental sessions. The first experimental session was conducted at least 48 hr after the initial screening. Both experimental sessions were completed within the same menstrual cycle, during the early follicular phase (0–7 days), or during the placebo (low hormone) phase if on hormonal contraceptives, both of which were verified by self‐report. Participants were required to refrain from engaging in strenuous exercise and consuming over‐the‐counter medications, including nonsteroidal anti‐inflammatory agents, vitamins, and minerals at least 48 hr prior to the sessions. In addition, they were restricted from consuming beverages containing caffeine and alcohol for at least 12 hr prior to the sessions. On the day of the experimental sessions, participants were allowed to consume food for up to 2 hr prior to the start of the session.

During the screening session, body mass was measured using a digital weight scale platform (model CBU150X; Mettler Toledo, Schwerzenbach, Switzerland) with a weighing terminal (model IND560; Mettler Toledo Inc., Mississauga, ON, Canada). Height was assessed using an eye‐level physician stadiometer (Detecto, model 2391, Webb City, MO, USA). Measured weight and height were used to calculate body surface area using the DuBois & DuBois equation (DuBois & DuBois, 1916). Body fat percentage was estimated through body density via the hydrostatic weighing technique. VO_2peak_was determined via an incremental cycling exercise protocol. Participants were seated on a semirecumbent cycling ergometer and were asked to maintain a consistent pedaling rate between 60 and 100 rpm. The resistance was set to 60 W, which was then increased by 20 W/min until volitional fatigue. Ventilation and metabolic data were collected with an automated indirect calorimetry system (Medgraphic Ultima; Medical Graphic, St. Paul, MN, USA).

### 2.4 Protocol 1: exercise‐heat stress

This protocol was identical to our previous study examining the modulating effect of HSP90 on cutaneous vasodilation in young men throughout rest, exercise, and recovery in the heat (Fujii, Zhang, et al., 2017). Upon arrival to the laboratory, participants voided their bladder, after which nude body mass was measured on a weighing terminal (Mettler Toledo Inc.). Following the measurement of nude body mass, participants were seated on a reclining medical bed in a nonheat stress environment (~25°C). Four microdialysis fibers (30 kDa cutoff, 10 mm membrane) (MD2000, Bioanalytical Systems, West Lafayette, IN, USA) were inserted into the dermal layer of the skin on the left dorsal forearm. Under aseptic conditions, a 25‐gauge needle was inserted into the skin (~2.5 cm in length), then the microdialysis fiber was threaded through the lumen of the needle. Thereafter, the needle was removed from the skin, leaving the fiber in place. Each fiber was secured with surgical tape, and separated by at least 2 cm. After the insertion of the fibers, the participants were transferred to an adjacent thermal chamber (Can‐Trol Environmental Systems, Markham, ON, Canada) regulated at 25°C and 20% relative humidity (RH) where they remained resting while seated on a semirecumbent cycle ergometer. At this time, the perfusion of the pharmacological agents was started at each of the four microdialysis sites at a rate of 4 μL min^−1^ with a micro‐infusion pump (Model 400, CMA Microdialysis, Solna, Sweden). The sites were perfused with either (a) lactated Ringers solution (Baxter, Deerfield, IL, USA) (control), (b) 178 μM Geldanamycin (Cayman Chemical (Ann Arbor, MI, USA), an inhibitor of HSP90, (c) 10 mM *N* ^G^‐nitro‐L‐arginine methyl ester (L‐NAME, Sigma‐Aldrich (St. Louis, MO, USA), a nonspecific NOS inhibitor, or (d) a combination of 178 μM Geldanamycin and 10 mM L‐NAME (HSP90 and NOS inhibition). A 5% dimethyl sulfoxide solution (Sigma‐Aldrich) was used to dissolve Geldanamycin. To offset any potential influence of dimethyl sulfoxide on responses, all of the above agents were dissolved in 5% dimethyl sulfoxide solution. Previously, we reported that 5% dimethyl sulfoxide solution had no effect on the cutaneous vascular response during exercise in the heat (Fujii, Zhang, et al., 2017). Concentrations of Geldanamycin (Shastry & Joyner, 2002a) and L‐NAME (Craighead, McCartney, Tumlinson, & Alexander, 2017; Fujii, Meade, et al., 2017; Houghton, Meendering, Wong, & Minson, 2006; Kellogg, Zhao, Coey, & Green, 2005; Wong, Wilkins, Holowatz, & Minson, 2003; Yamazaki, Takahara, Sone, & Johnson, 2007) were determined based on previous studies. The drug perfusion continued for 90 min to maximize inhibition of HSP90 and NOS. Further, this time period has been shown to be sufficient to ensure that trauma associated with fiber insertion had subsided (Anderson, Andersson, & Wardell, 1994). To ensure the continuous inhibition of HSP90 and NOS, the drug perfusion was continued throughout the entire experimental protocol.

Following a minimum 60min resting period under a normothermic nonheat stress condition (i.e., room temperature of 25°C and RH of 20%), a 10 min baseline measurement was taken. Thereafter room temperature was elevated rapidly to 35°C (RH 20%) during which time the participants rested for at least 70 min, with the last 10 min of this period used for data analysis (preexercise resting). Participants then cycled for 50 min at a moderate intensity equivalent to ~55% of their predetermined VO_2peak_ followed by 30 min of recovery. Thereafter, 50 mM ofsodium nitroprusside (Sigma‐Aldrich) was infused for 20–25 min at all four sites at a rate of 6 μL min^−1^ until maximum values of cutaneous perfusion were obtained for a minimum of 3 min. Upon completion of the experimental session, the participants’body mass was measured.

### 2.5 Protocol 2: passive whole‐body heat stress

Participants rested in a semirecumbent position in a thermoneutral room (~25°C) while wearing a tube‐lined water‐perfusion suit that was continuously perfused with water at a temperature of 34°C. Four microdialysis fibers were then inserted intradermally on the uncovered dorsal forearm and the sites were perfused with the same agents according to the procedures described for Protocol 1. Following the 90min resolution period, a 10min baseline resting measurement period was performed, after which the temperature of the water perfusing the suit was increased to 49.5°C to initiate an increase in body core temperature (as estimated by rectal temperature, see below) of ~1.0°C, which was equivalent to the mean increase in body core temperature at end‐exercise. To minimize sweat evaporation (which cools the body) and induce a gradual and progressive increase in body core temperature, the suit was also covered in a plastic sheet and blanket. As with the exercise‐heat stress protocol, the perfusion of each microdialysis fiber was maintained throughout passive heating. Once body core temperature had reached the target level of ~1.0°C above baseline, the suit water temperature was reduced to 43°C to allow for a stable plateau in cutaneous vascular conductance to develop (10 min). Heating lasted for 60–90 min to allow body core temperature to increase and stabilize at ~1.0°C above baseline. Thereafter, the maximum absolute CVC was determined as described in Protocol 1.

### 2.6 Measurements

An index of cutaneous blood flow, laser‐Doppler flux expressed in perfusion units, was measured at four local sites on the forearm using laser‐Doppler flowmetry (PeriFlux System 5000, Perimed, Stockholm, Sweden) at a sampling rate of 32 Hz. Integrated seven‐laser array laser‐Doppler probes (Model 413, Perimed, Stockholm, Sweden) were positioned directly over the center of the membrane of the microdialysis fibers. Cutaneous vascular conductance (CVC) was evaluated as laser‐Doppler flux divided by mean arterial pressure, and expressed as a percentage of the maximum recorded value (i.e., CVC_%max_) using values from the maximal absolute CVC protocol. Blood pressure was determined every 5 min using a manual mercury column sphygmomanometer (Baumonometer Standby Model, WA Baum Co, Copiague, NY, USA). Mean arterial pressure was calculated as diastolic arterial pressure plus one‐third the difference between systolic and diastolic pressures.

Rectal temperature was measured as an index of body core temperature for both protocols with a pediatric thermocouple probe ~2 mm in diameter (Mon‐a‐therm, Mallinckrodt Medical, MO, USA) that was self‐inserted ~12 cm beyond the anal sphincter. Skin temperature was measured at four sites, namely the chest, biceps, quadriceps, and calf using thermocouple discs (Concept Engineering, Old Saybrook, CT, USA) attached to the skin with adhesive rings and surgical tape. Mean skin temperature was then estimated as a weighted mean using the local skin temperatures of the chest (30%), biceps (30%), quadriceps (20%), and calf (20%) (Hardy & Dubois, 1938). Temperature data were collected at 15‐s intervals using a data acquisition model (Model 34970A; Agilent Technologies Canada Inc., Mississauga, ON, Canada) and displayed and recorded using LabVIEW software (National Instruments, Austin, TX, USA). Heart rate was measured continuously using a Polar coded WearLink and transmitter, Polar RS400 interface, and stored using the Polar Trainer 5 software (Polar Electro, Kempele, Finland).

During exercise, metabolic energy expenditure was measured continuously using electrochemical gas analyzers (AMETEK model S‐3A/1 and CD3A, Applied Electrochemistry, Pittsburgh, PA, USA) to determine O_2_ and CO_2_ concentrations in expired air. Participants were fitted with a facemask (Model 7600 V2, Hans‐Rudolph, Kansas City, MO, USA), that was attached to a 2‐way T‐shape nonbreathing valve (Model 2700, Hans‐Rudolph). The mask was worn during the first 10–15 min of exercise to verify the exercise work‐load. Oxygen uptake and respiratory exchange ratio were calculated from O_2_ and CO_2_ concentrations in expired air, and sampled every 30 s to estimate metabolic rate.

Prior to each experimental protocol, urine specific gravity (an index of hydration status) was evaluated using a handheld total solids refractometer (Model TS400, Reichter Inc., Depew, NY).

### 2.7 Data analysis

Based on CVC_%max_ data from our previous work, a minimum sample size of *n* = 10 was required, with Power = 80% and α = .05. For Protocol 1, CVC_%max_ values were presented as the mean of the final 5 min of each 10‐min measurement period for rest under nonheat stress (25°C) and heat stress (35°C) and for every 10 min throughout exercise and recovery. For Protocol 2, CVC_%max_ values were presented for the final 5 min of each 10‐min measurement period for baseline and the plateau phase of whole‐body heating (+1.0°C in body core temperature). To determine the magnitude of increase in CVC_%max_ for a given increase in body core temperature, the final 1 min of each 0.1°C increase in body core temperature from 0.0°C to 1.0°C was presented. For both protocols, 5‐min average values for temperature and heart rate responses are presented for the respective baseline (Protocol 1: rest at 35°C; Protocol 2: 0°C increase in body core temperature) and plateau (Protocol 1: end‐exercise; Protocol 2:1.0°C increase in rectal temperature) periods. Blood pressure was manually recorded every 5 min, and presented as the average of two values measured during each period. For both protocols, maximum CVC was presented as the highest 3min average during sodium nitroprusside infusion. For Protocol 1, one control site was removed for a participant and one HSP90 inhibited site was removed for another participant due to technical issues. For Protocol 2, one control site was removed for a participant and one NOS inhibited site was removed for another participant due to technical issues. Additionally, mean skin temperature was not recorded for one participant in Protocol 2 due to technical issues.

### 2.8 Statistical analyses

For Protocol 1, CVC_%max_ was analyzed with a linear mixed model with the factors of treatment site (control, HSP90 inhibition, NOS inhibition, combined HSP90 and NOS inhibition) and measurement period (baseline rest and each 10 min interval during the 50min exercise and subsequent 30min postexercise recovery in the heat (35°C)). For Protocol 2, CVC_%max_ was analyzed with a linear mixed model with the factors of treatment site (as above) and change in body core temperature from baseline (every 0.1°C increase in body core temperature from 0.0°C to 1.0°C, and plateau). To directly compare both models of heat stress, CVC_%max_ responses were evaluated between end‐exercise in Protocol 1, where a significant effect of HSP90 was observed (see results below), and the matched change in body core temperature for each participant during passive whole‐body heating in Protocol 2. A linear mixed model with the factors of treatment site (as above) and protocol (exercise and passive whole‐body heating) was used to compare responses between protocols. Maximum CVC (perfusion units·mmHg^−1^) was analyzed with a linear mixed model with the factors of protocol (as above) and treatment site (as above). Urine specific gravity was compared between protocols using a two‐way paired *t*‐test. All other secondary variables (core and mean skin temperatures, heart rate, and mean arterial pressure) were analyzed with a linear mixed model with the factors of protocol (as above) and measurement period (baseline and plateau heating). After detecting a significant main effect or interaction, *post hoc* multiple comparisons were carried out using either Tukey's test or the Bonferroni procedure where appropriate. The level of significance for all analyses was set at *p* < .05. All values are reported as mean ± 95% confidence interval (1.96 × standard error of the mean) or mean ± standard deviation, where appropriate. Statistical analyses were conducted using GraphPad Prism v.8.4.1 (GraphPad, CA, USA).

## 3 RESULTS

### 3.1 Hydration status

No differences in pretrial urine specific gravity were noted between the exercise‐heat stress (USG: 1.011 ± 0.009) and passive whole‐body heating (1.008 ± 0.007) protocols (*p* > .05). All participants were euhydrated prior to beginning each protocol.

### 3.2 Body temperatures and cardiovascular responses

There was a significant main effect of measurement period for body core temperature (*p* < .001), which was increased from baseline (37.29 ± 0.34°C) to end‐exercise (38.30 ± 0.36 °C) during Protocol 1 and from baseline (37.28 ± 0.26°C) to plateau (38.35 ± 0.32°C) during Protocol 2 (both, *p* < .05). There was a significant protocol by measurement period interaction for mean skin temperature (*p* < .001), which was increased from baseline (33.65 ± 0.71 °C) to end‐exercise (35.32 ± 0.39°C) during Protocol 1 and from baseline (34.03 ± 0.47°C) to plateau (37.04 ± 0.72 °C) during Protocol 2 (both *p* < .05). There was a significant protocol by measurement period interaction for heart rate (*p* < .001), which was increased from baseline (77 ± 17 bpm) to end‐exercise (154 ± 13 bpm) during Protocol 1 and from baseline (63 ± 8 bpm) to plateau (100 ± 12 bpm) during Protocol 2 (both *p* < .05). There was a significant protocol by measurement period interaction for mean arterial pressure (*p* = .035), which was increased from baseline (89 ± 6 mmHg) to end‐exercise (97 ± 15 mmHg) during Protocol 1 (*p* < .05) but not from baseline (86 ± 10 mmHg) to plateau (85 ± 11 mmHg) during Protocol 2 (*p* > .05).

### 3.3 Cutaneous vascular conductance

#### 3.3.1 Exercise‐heat stress (Protocol 1)

**FIGURE 1:**
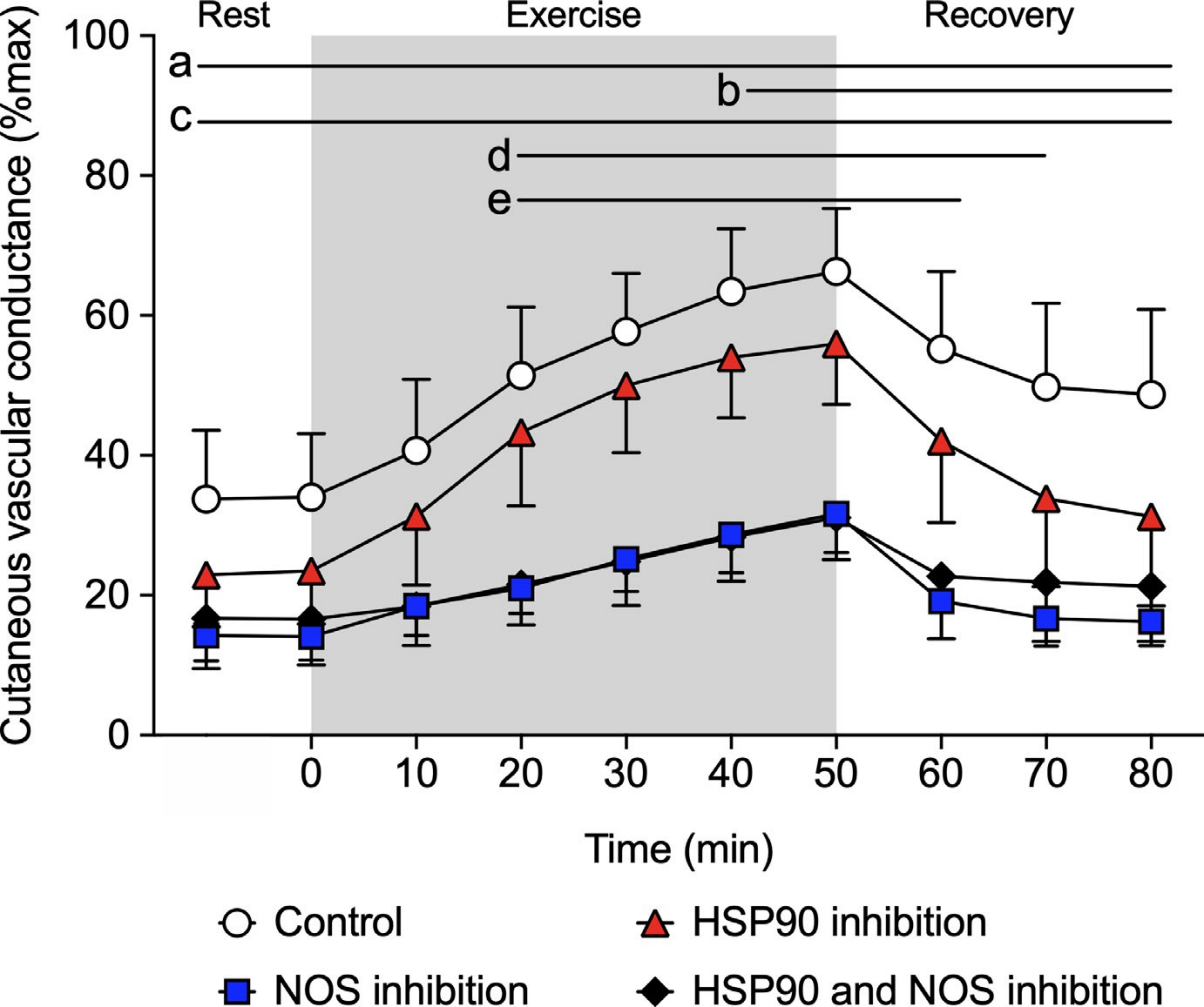
Cutaneous vascular conductance (%max) during rest, exercise and recovery in the heat (35°C). Responses were evaluated at four dorsal forearm skin sites that were continuously treated with: (1) lactated Ringer solution (control, white circles), (2) 178 μM Geldanamycin (heat shock protein 90 (HSP90) inhibition, red triangles), (3) 10 mM L‐NAME (nitric oxide synthase (NOS) inhibition, blue squares), or (4) combined 178 μM Geldanamycin and 10 mM L‐NAME (HSP90 and NOS inhibition, black diamonds), via intradermal microdialysis. Data are presented as mean ± 95% confidence interval (*n* = 11). ^a^NOS inhibition versus control (*p* < .05); ^b^HSP90 inhibition versus control (*p* < .05); ^c^HSP90 and NOS inhibition combined versus control (*p* < .05); ^d^NOS inhibition versus HSP90 inhibition (*p* < .05); ^e^HSP90 and NOS inhibition combined versus HSP90 inhibition alone (*p* < .05). There was a significant treatment site by time interaction (*p* < .001)

There was a significant treatment site by time interaction for CVC_%max_ (*p* < .001, Figure 1). During rest in the heat (35°C) NOS inhibition alone and combined HSP90 and NOS inhibition attenuated CVC_%max_ relative to control, but not relative to HSP90 inhibition alone (both *p* > .05), which persisted throughout exercise and recovery (all *p* < .05). The attenuation of CVC_%max_ relative to control with NOS inhibition alone, and with combined HSP90 and NOS inhibition, persisted throughout exercise and recovery (all *p* < .05). Inhibition of HSP90 alone attenuated CVC_%max_ relative to control from 40 min into exercise until the end of recovery (all *p* < .05).

#### 3.3.2 Passive‐heat stress (Protocol 2)

**FIGURE 2:**
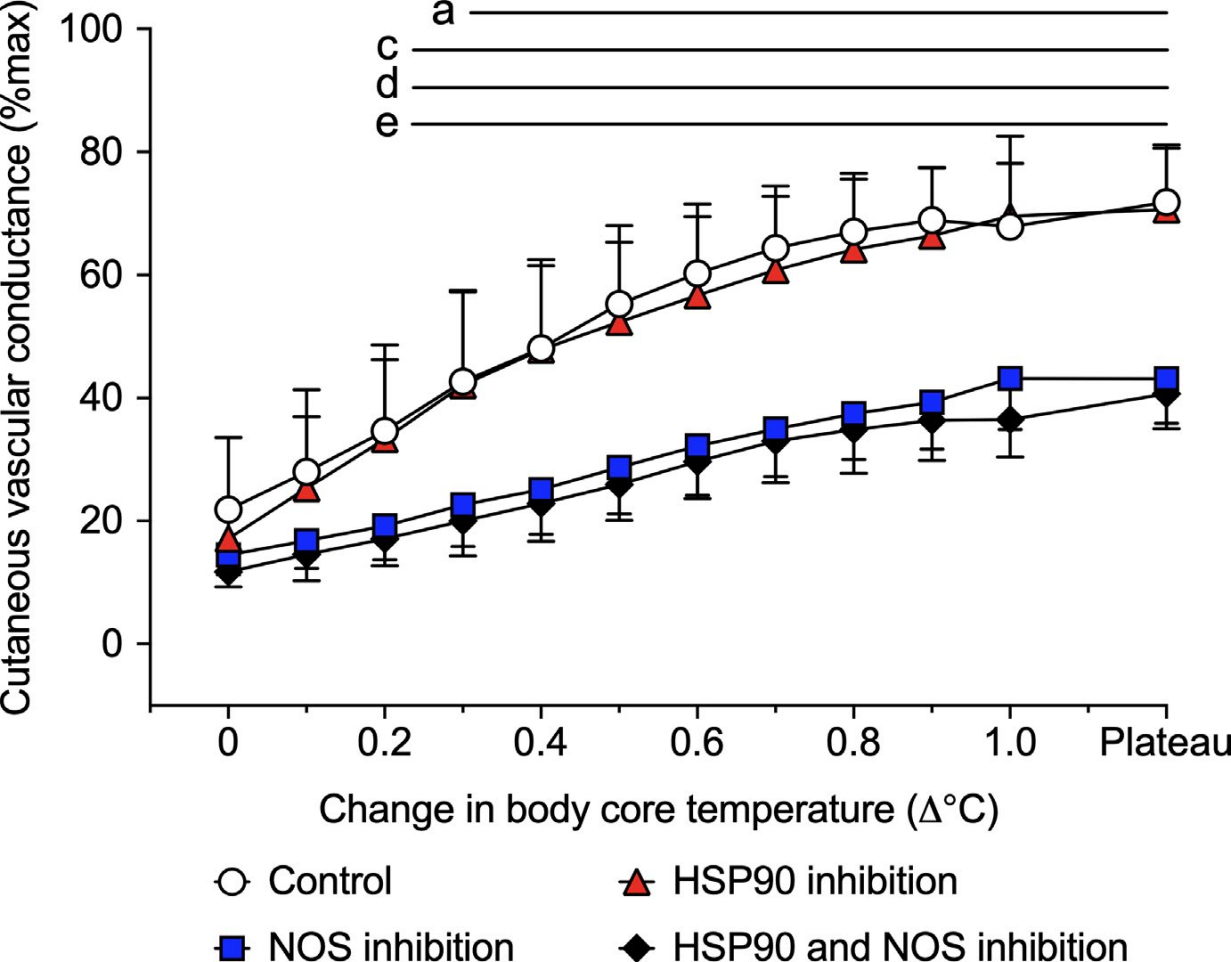
Cutaneous vascular conductance (%max) during passive whole‐body heating. Responses were evaluated at four dorsal forearm skin sites that were continuously treated with: (1) lactated Ringer solution (control, white circles), (2) 178 μM Geldanamycin (heat shock protein 90 (HSP90) inhibition, red triangles), (3) 10 mM L‐NAME (nitric oxide synthase (NOS) inhibition, blue squares), or (4) combined 178 μM Geldanamycin and 10 mM L‐NAME (HSP90 and NOS inhibition, black diamonds), via intradermal microdialysis. Data are presented as mean ± 95% confidence interval (*n* = 11). ^a^NOS inhibition versus control (*p* < .05); ^b^HSP90 inhibition versus control (*p* < .05); ^c^HSP90 and NOS inhibition combined versus control (*p* < .05); ^d^NOS inhibition versus HSP90 inhibition (*p* < .05); ^e^HSP90 and NOS inhibition combined versus HSP90 inhibition alone (*p* < .05). There was a significant treatment site by body core temperature interaction (*p* = .002)

There was a significant treatment site by body core temperature interaction for CVC_%max_ (*p* = .002, Figure 2). While CVC at all skin sites progressively increased along with elevations in body core temperature, HSP90 inhibition alone did not alter CVC_%max_ relative to control (all *p* > .05). Conversely, combined HSP90 and NOS inhibition, and NOS inhibition alone attenuated CVC_%max_ relative to control from 0.2°C and 0.3°C above baseline, respectively until the plateau phase of whole‐body heating (all *p* < .05).

#### 3.3.3 Exercise‐ versus passive‐heat stress

**FIGURE 3:**
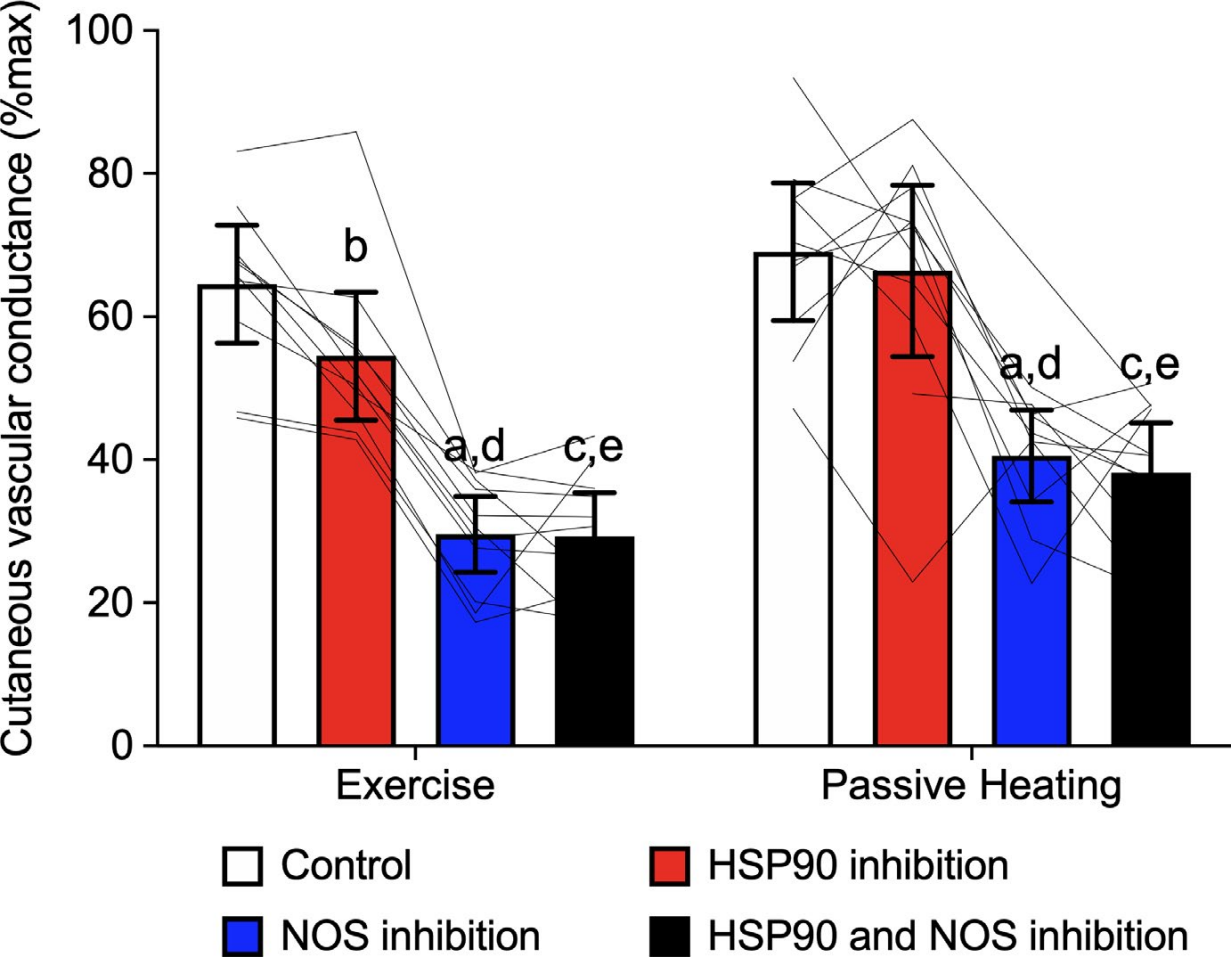
Comparison of cutaneous vascular conductance (%max) between end‐exercise in the heat and the matched elevation in body core temperature from baseline during passive whole‐body heating. Responses were evaluated at four dorsal forearm skin sites that were continuously treated with: (1) lactated Ringer solution (control, white bars), (2) 178 μM Geldanamycin (heat shock protein 90 (HSP90) inhibition, red bars), (3) 10 mM L‐NAME (nitric oxide synthase (NOS) inhibition, blue bars), or (4) combined 178 μM Geldanamycin and 10 mM L‐NAME (HSP90 and NOS inhibition, black bars), via intradermal microdialysis. Group data are presented as mean ± 95% confidence interval (*n* = 11). Individual responses are presented as black lines within each protocol. ^a^NOS inhibition versus control (*p* < .05); ^b^HSP90 inhibition versus control (*p* < .05); ^c^HSP90 and NOS inhibition combined versus control (*p* < .05); ^d^NOS inhibition versus HSP90 inhibition (*p* < .05); ^e^HSP90 and NOS inhibition combined versus HSP90 inhibition alone (*p* < .05). There were significant main effects for treatment site (*p* < .001) and protocol (*p* = .024)

When comparing CVC_%max_ between end‐exercise and the matched elevation in body core temperature during passive heating there were significant main effects of protocol (*p* = .024) and treatment site (*p* < .001), but not for the protocol by treatment site interaction (*p* > .05) (Figure 3). Overall, CVC_%max_ responses were higher during passive heating compared to end‐exercise despite matched increases in the level of hyperthermia, as defined by similar body core temperatures (i.e., +1.0°C above baseline resting) between protocols.

Maximum CVC achieved with sodium nitroprusside infusion did not differ between sites (*p* > .05, Table 1). However, there was a main effect of protocol such that maximum CVC responses were higher during the exercise protocol compared to the passive whole‐body heating condition (*p* < .001, Table 1).

**TABLE 1:** Maximum cutaneous vascular conductance

	Cutaneous vascular conductance (perfusion units·mmHg^−1^)			
	Control	HSP90 inhibition	NOS inhibition	HSP90 + NOS inhibition
Exercise heating	2.51 ± 0.98 (*n* = 10)	2.39 ± 0.61 (*n* = 10)	2.11 ± 0.50 (*n* = 11)	2.33 ± 0.74 (*n* = 11)
Passive heating	1.74 ± 0.49 (*n* = 10)	1.80 ± 0.26 (*n* = 11)	1.62 ± 0.54 (*n* = 10)	1.68 ± 0.25 (*n* = 11)

Note

All values are presented as mean ± standard deviation. There was a significant main effect of protocol (*p* = .0004) such that cutaneous vascular conductance responses were higher during exercise than during passive whole‐body heating.

## 4 DISCUSSION

This study is the first to examine the role of HSP90 in regulating cutaneous vasodilation in young women during both exercise‐ and passive‐heat stress. Consistent with our first hypothesis, HSP90 attenuated cutaneous vasodilation via a NOS‐dependent mechanism when body core temperature was elevated near end‐exercise (final 10 min of the 50min exercise bout). In contrast to our second hypothesis, HSP90 inhibition did not influence cutaneous vasodilation during passive heating up to and including a body core temperature elevation of 1.0°C. This was evident, despite NOS contributing to cutaneous vasodilation throughout passive heating. Consequently, in contrast to our third hypothesis, the HSP90 contribution during end‐exercise, which attenuated cutaneous vasodilation, was not consistent with that of passive heating for the same elevation in body core temperature, where a negligible effect was observed. Taken together, we show that HSP90 modulates cutaneous vasodilation in young women during an exercise‐heat stress via a NOS‐dependent mechanism, which was not solely related to the elevation in body core temperature, as determined by a negligible HSP90 contribution during passive heating.

### 4.1 Exercise‐heat stress

In young women, while HSP90 did not influence CVC_%max_ during rest in the heat (35°C), NOS inhibition and combined HSP90 and NOS inhibition both attenuated CVC_%max_ under this condition. At exercise onset, CVC_%max_ at the control site increased from pre‐exercise resting levels in the heat, reaching ~66% of maximum at end‐exercise, along with elevations in body core temperature. Importantly, HSP90 inhibition did not influence CVC_%max_ during the early phase of exercise when nonthermal factors such as central command, mechanoreflex, baroreceptors, and others are known to play an important role in modulating cutaneous vasodilation and therefore heat dissipation (Kenny & Jay, 2013). However, we showed that HSP90 inhibition attenuated CVC_%max_ relative to the control site (10%–11%) during the latter phase of exercise (40–50 min) when there was a pronounced elevation in body core temperature. These effects are comparable to our previous observations in young men wherein HSP90 inhibition also attenuated CVC_%max_ during the latter phase of exercise (30–50 min) relative to the control site by 15%–20% (Fujii, Zhang, et al., 2017). Further in line with our prior findings in young men, here we demonstrate that HSP90 modulates cutaneous vasodilation in young women during moderate‐intensity exercise in the heat by interacting with NOS. This was demonstrated by the observation that the HSP90 contribution to cutaneous vasodilation during exercise was abolished when NOS was simultaneously inhibited.

During postexercise recovery, CVC_%max_ at the control site declined rapidly, yet remained elevated above preexercise rest in the heat. However, the HSP90 contribution to cutaneous vasodilation was sustained throughout recovery in the heat in the young women. This contrasts with our previous finding in young men wherein the HSP90 contribution to cutaneous vasodilation was abolished within the first 10 min of recovery (Fujii, Zhang, et al., 2017). This discrepancy may be due to sex differences in the withdrawal of cutaneous vasodilation during postexercise recovery associated with the possible modulating influence of nonthermal factors (Kenny & Jay, 2013). However, more work is required to elucidate the mechanisms underpinning the HSP90 contribution to cutaneous vasodilation during postexercise recovery and the potential sex differences that mediate this response.

### 4.2 Passive‐heat stress

As anticipated, passive heating increased CVC_%max_ relative to baseline. Further, NOS inhibition alone or in combination with HSP90 inhibition progressively attenuated CVC_%max_ relative to the control site as body core temperature increased. However, unlike during exercise in the heat, HSP90 inhibition alone did not attenuate CVC_%max_ relative to the control site even for the same elevation in body core temperature as end‐exercise where a clear modulating effect of HSP90 on cutaneous vasodilation was observed. Indeed, individual responses at end‐exercise demonstrated a fairly consistent attenuating effect of HSP90 on CVC_%max_, whereas no such relationship was evident during passive heating (Figure 3). Combined, these findings indicate that HSP90 does not modulate cutaneous vasodilation during passive whole‐body heating either through NOS‐dependent or independent pathways.

We previously surmised that HSP90 activation during exercise may be related to acetylcholine released from sympathetic nerves (Fujii, Zhang, et al., 2017). Indeed, Shastry and Joyner demonstrated that acetylcholine‐induced cutaneous vasodilation was in part due to HSP90 activation (Shastry & Joyner, 2002b). Passive whole‐body heat stress activates sympathetic cholinergic nerves that in turn release acetylcholine, contributing to cutaneous vasodilation (Kellogg et al., 1995; Shibasaki, Wilson, Cui, & Crandall, 2002). However, the negligible influence of HSP90 in modulating cutaneous vasodilation during passive heating in this study indicates that acetylcholine released from sympathetic nerves does not play an important functional role in mediating cutaneous vasodilation in vivo. Although direct confirmation of this during an exercise‐heat stress would need to be examined by using a simultaneous acetylcholine receptor blocker such as atropine.

### 4.3 Exercise‐ versus passive‐heat stress

It is well established that the NOS isoforms regulating cutaneous vasodilation differ based on the mode of heat stress. While endothelial NOS primarily modulates cutaneous vasodilation during local heating (Kellogg, Zhao, & Wu, 2008b; Kellogg et al., 2009) and an exercise‐heat stress (Fujii et al., 2016; McNamara et al., 2014), neuronal NOS mainly regulates this response during passive whole‐body heating (Kellogg, Zhao, & Wu, 2008a; Kellogg et al., 2009; McNamara et al., 2014). A clear link has been shown between HSP90 and endothelial NOS activity in both animal and in vitro work (Harris, Blackstone, Ju, Venema, & Venema, 2003; Lin, Lin, Ho, & Liau, 2005; Ou et al., 2004). However, HSP90 has also been shown to interact directly with neuronal NOS in human coronary artery smooth muscle (Han et al., 2009). Based on our current and previous work demonstrating an attenuating effect of HSP90 inhibition on CVC_%max_ during exercise‐heat stress (Fujii, Zhang, et al., 2017), it is plausible that HSP90 primarily contributes to cutaneous vasodilation under these conditions by stimulating endothelial NOS. In support of this, HSP90 also modulates the endothelial NOS‐dependent vasodilatory response associated with sustained local heating in human skin (Shastry & Joyner, 2002b). Further, the negligible contribution of HSP90 to cutaneous vasodilation during passive whole‐body heating in this study, despite a clear NOS contribution, strongly indicates that this protein does not functionally interact with neuronal NOS in human skin. However, this needs to be confirmed using specific receptor blockers for each NOS isoform in conjunction with HSP90 inhibition to evaluate cutaneous vasodilator responses during both forms of heat stress.

### 4.4 Perspective and significance

Cutaneous vasodilation is a critical avenue for heat dissipation and therefore the regulation of body core temperature during exercise, especially in the heat. It is well established that NOS plays an important role in modulating this heat loss response in young adults and sex‐dependent differences in NOS‐dependent cutaneous vasodilation have been shown to exist (Stanhewicz et al., 2014). Our current and previous work indicates that the contribution of HSP90 in mediating NOS‐dependent cutaneous vasodilation is similar for women and men. While the young women in this study were all tested during the early follicular (low hormone) phase of their menstrual cycles, the cutaneous vasodilator response to heating is augmented during the high hormone phase (Charkoudian, Stephens, Pirkle, Kosiba, & Johnson, 1999). Estrogen is an important modulator of NOS, the effects of which may be influenced by menstrual cycle phase in association with fluctuations in the circulating concentration of this sex hormone (Hayashi et al., 1995). Further, HSP90 plays an important role in estrogen‐mediated modulation of endothelial NOS activity in both porcine (Schulz, Anter, Zou, & Keaney, 2005) and human (Russell et al., 2000) vascular endothelial cells. Combined, these findings indicate that circulating estrogen may influence the relative contribution of HSP90 to cutaneous vasodilation in young women in vivo. As such, future work should evaluate the effects of menstrual cycle phase on the NOS‐dependent contribution of HSP90 to cutaneous vasodilation during an exercise‐heat stress.

Finally, the disparate findings regarding the role of HSP90 in modulating cutaneous vasodilation in this study coincide with other work showing differences in the modulation of this heat loss response between the two heating modalities (Fujii et al., 2018; Holowatz et al., 2006; Kellogg et al., 2009; McCord et al., 2006; McNamara et al., 2014; Meade et al., 2019). This highlights the need to further explore the end‐organ factors that modulate cutaneous vasodilation during exercise‐heat stress in healthy young adults, since this response remains poorly understood relative to that of passive whole‐body heating and it is becoming increasingly clear that one model cannot readily be used as a surrogate to inform the other.

## 5 CONCLUSION

We showed that in young women, HSP90 contributes to cutaneous vasodilation during the latter phase of steady‐state exercise and recovery in the heat and that this effect is NOS‐dependent. Conversely, HSP90 did not contribute to cutaneous vasodilation during passive whole‐body heat stress, even at the same elevated level of body core temperature as the exercise‐heat stress protocol where a modulating effect on cutaneous vasodilation was observed. This highlights the need for further work exploring the factors that modulate cutaneous vasodilation during exercise‐heat stress in young women.

## CONFLICT OF INTEREST

None declared.

## AUTHOR CONTRIBUTIONS

G.W.M., N.F. and G.P.K. conceived and designed the experiments. G.W.M., C.M.M., and M.D.S. performed the data collection and analysis. G.W.M., N.F., and G.P.K. interpreted the results. G.W.M. prepared figures and drafted the manuscript. All authors edited and revised the manuscript. All authors approved the final version of the manuscript. All experiments took place at the Human and Environmental Physiology Research Unit, located at the University of Ottawa.
